# Determination of Ignitable Liquids in Fire Debris: Direct Analysis by Electronic Nose

**DOI:** 10.3390/s16050695

**Published:** 2016-05-13

**Authors:** Marta Ferreiro-González, Gerardo F. Barbero, Miguel Palma, Jesús Ayuso, José A. Álvarez, Carmelo G. Barroso

**Affiliations:** 1 Department of Analytical Chemistry, Faculty of Sciences, IVAGRO, University of Cadiz, Campus Universitario, 11510 Puerto Real, Spain; marta.ferreiro@uca.es (M.F.-G.); gerardo.fernandez@uca.es (G.F.B.); carmelo.garcia@uca.es (C.G.B.); 2 Department of Physical Chemistry, Faculty of Sciences, University of Cadiz, P.O. Box 40, 11510 Puerto Real, Cádiz, Spain; jesus.ayuso@uca.es (J.A.); joseangel.alvarez@uca.es (J.A.Á.)

**Keywords:** fire accelerants, discrimination, optimization, E-Nose

## Abstract

Arsonists usually use an accelerant in order to start or accelerate a fire. The most widely used analytical method to determine the presence of such accelerants consists of a pre-concentration step of the ignitable liquid residues followed by chromatographic analysis. A rapid analytical method based on headspace-mass spectrometry electronic nose (E-Nose) has been developed for the analysis of Ignitable Liquid Residues (ILRs). The working conditions for the E-Nose analytical procedure were optimized by studying different fire debris samples. The optimized experimental variables were related to headspace generation, specifically, incubation temperature and incubation time. The optimal conditions were 115 °C and 10 min for these two parameters. Chemometric tools such as hierarchical cluster analysis (HCA) and linear discriminant analysis (LDA) were applied to the MS data (45–200 *m*/*z*) to establish the most suitable spectroscopic signals for the discrimination of several ignitable liquids. The optimized method was applied to a set of fire debris samples. In order to simulate post-burn samples several ignitable liquids (gasoline, diesel, citronella, kerosene, paraffin) were used to ignite different substrates (wood, cotton, cork, paper and paperboard). A full discrimination was obtained on using discriminant analysis. This method reported here can be considered as a green technique for fire debris analyses.

## 1. Introduction

In many arson cases accelerants such as ignitable liquids are used to initiate or accelerate a fire. Therefore, the detection of ignitable liquid residues (ILR) at fire scenes is a key step in any fire investigation [1]. The identity of the type of accelerant is consequently very useful information for investigators in cases where arson is suspected. The most commonly used ignitable liquids are petroleum-based products like gasoline and diesel as they are easy to obtain and easy to ignite [2]. When investigators attempt to determine the cause of a fire, they usually start with an analysis of the fire debris in order to identify some residues from any ignitable liquid used to start the fire.

Gas chromatography-mass spectrometry is the most widely used analytical technique for the determination of accelerants in fire debris. The American Society for Testing and Materials International (ASTM International) standard provides guidelines for the identification and classification of ILRs from fire debris by GC-MS [3]. The National Center for Forensic Science maintains a large online database of GC-MS data for ignitable liquids [4].

Prior to chromatographic analysis it is a requirement that the ignitable liquid residue is in a vapor or volatile liquid form. As a consequence, a suitable sample preparation step for the ILR from fire debris samples is usually necessary. Different sample preparation standard practices have been approved by the ASTM to isolate the ILR. Standard Practice for Separation of Ignitable Liquid Residues from Fire Debris Samples by Passive Headspace Concentration With Activated Charcoal, ASTM E1412, [5] is currently the most commonly used method in the U.S. to isolate and concentrate ILRs from fire debris because it is sensitive, easy to operate and it is non-destructive [6,7] whilst SPME using Tenax as sorbent is the most used technique in European countries. The ASTM E1412 based in the extraction with an active charcoal strip, which is carried out for 12 to 16 h between 60 and 90 °C. Although this method works well, it has the drawback of requiring the use of a solvent to extract volatile compounds from the activated charcoal followed by injection of the solvent into the GC system. Besides, the most widely used solvent is carbon disulfide because it has proven to be the most efficient, although it is highly toxic and has a very low autoignition temperature, around 100 °C, thus making it dangerous even around the boiling point of water [8].

As a consequence, other absorbents such as Tenax or Carbotrap, which can be thermally desorbed, have also been studied [9]. Recently, St. Pierre *et al.* proposed and evaluated a novel methodology designed to optimize the recovery of oxygenated ignitable liquids from fire debris samples using zeolites as an adsorbent medium [10]. In order to extract the ILRs with zeolites, samples need to be heated for 4 h at 120 °C. Although this method is faster than the ASTM E1412 standard method and the zeolites are very cheap, much cheaper than the activated charcoal strips, it also requires the use of solvent (MeOH) and can only be applied to oxygenated ignitable liquids. Another study was carried out in which the authors compared the recoveries of petroleum and alcohol-based ignitable liquid mixtures using activated charcoal strips and zeolites, both individually and in tandem [11]. The best results were obtained using zeolites with activated charcoal strips in a ‘dual-mode’. Samples in this case were heated for 2 h at 85 °C–90 °C and the method requires the use of solvents (MeOH and CS_2_), additional adsorbent preparation, and a second GC/MS run for the zeolite extract.

SPME is also used since it is highly sensitive, nondestructive, rapid, and it does not require the use of solvents [12,13,14,15,16]. SPME could be a good alternative to activated charcoal strips (ACS) and, in fact, the ASTM recommends SPME as a screening test for fire debris analysis. However, this method also suffers from some drawbacks: the low robustness of the fibers, their limited useful life, and their cost *versus* activated charcoal. Furthermore, while the extraction time is minimal, it is more difficult to automate [1,17]. A different method for the preconcentration of combustion accelerants has been developed by Cacho *et al.* They have presented a novel method based on headspace sorptive extraction (HSSE) for the isolation and identification of ILs in fire debris [17]. Recently, porous layer open tubular (PLOT) columns have been also used for fire debris analysis. Nichols *et al.* [18] used PLOT with cryoadsorption to determine ignitable liquids in fire debris. PLOT-cryoadsorption provided a larger number of compounds and faster analysis time than the regular purge and trap systems. Additionally, PLOT-cryoadsorption produced lower recoveries for the most volatiles compounds than the activated charcoal strips method, however using only 3 min instead of 2 h. Regardless of the preconcetration method, the analysis of the ignitable liquids is performed by gas chromatography. According to the ASTM E1618 standard the identification of the presence of an ignitable liquid residue in fire debris samples relies on visual pattern recognition of the total ion chromatogram (TIC), extracted ion chromatograms (EIC), and target compound analysis in order to identify the presence of an ignitable liquids residue in fire debris samples.

Although this method works well, it is time consuming, interpretation of the results is highly dependent on the experience of the analyst, and it does not allow automation. Therefore, the use of chemometric tools is almost mandatory to help the analyst to identify and classify accelerants easily in a shorter time. Furthermore, this approach does not consider certain factors that may alter the chromatographic profile and hence complicate the classification, e.g., the type of substrate or the pyrolysis products, amongst others [19]. In order to provide the fire debris analyst with an automated database searching tool that minimizes laboratory-to-laboratory chromatography, some authors have proposed alternative methods to TIC. In this sense, Sigman *et al.* developed a covariance mapping method to group ignitable liquids belonging to the same ASTM classification [6,20]. Although this method allowed similar ignitable liquids to be grouped, it was computationally demanding when implemented as a database search application. The same authors later proposed the use of the total ion spectrum (TIS) calculated by summing the intensities of each nominal mass over all chromatographic times in a GC-MS analysis. This new approach provided enough information for the rapid identification of ignitable liquids in a database [21]. TIS combined with chemometric tools (HCA, LDA, QDA or SIMCA) has already been applied as a method to discriminate and classify fire debris samples as well as to predict classification error rates [7,22,23,24,25]. A great deal of analytical work has been carried out in an effort to minimize the use of solvents, thus reducing analysis time and/or increasing the automation of the data analysis using fingerprints.

In the work described here, an alternative analytical technique based on E-Nose with a MS detection system (headspace - mass spectrometry electronic nose) for the analysis of ignitable liquids in fire debris is presented. The pattern response obtained by E-Nose is the summed ion spectrum, which is similar to the TIS and is obtained in only a few minutes since chromatographic separation is not required. Moreover, the E-Nose technique was optimized for the analysis of fire debris samples without using any adsorbent to isolate the ILRs and using as the pre-concentration method the static headspace created in the autosampler oven of the E-Nose. The headspace is taken from the vial using a gas syringe and injected into the mass spectrometer without any chromatographic separation. Similar systems based on sensor array detection have previously been used for the initial evaluation of a fire scene [26,27]. However, the use of an array of sensors has several disadvantages such as poisoning, profile masking by some major constituents of the sample, the strong influence of moisture, and the non-linearity of signals [28]. All of these drawbacks are solved by using an MS detector.

The aforementioned technique has been applied in other fields [29,30,31,32,33]. Pavón *et al.* reviewed the main applications of HS-MS, also known as E-Nose, with special reference to applications in the environmental field [28]. Although most of the applications of HS-MS E-Nose to date have been related to the food and beverage area [32,33,34,35], similar systems have previously been applied for the discrimination of diesels [36] or for the analysis of methyl *tert*-butyl ether (MTBE) in gasoline samples [37]. 

Suitable chemometric treatment of the signal is essential in order to extract the information contained in the signal profile, as is the case for most non-separative methods. In a previous study, HS-MS E-Nose has been optimized and successfully applied for the identification of gasoline samples with different research octane numbers by using HCA and LDA [38,39] and for the thermal desorption of ACSs containing ILRs [40].

To our knowledge, the method proposed here is the first to be reported for direct analysis, *i.e.*, without extraction or adsorption steps, in fire debris investigation. The E-Nose experimental parameters were first optimized for the extraction of ILRs and then applied to the discrimination of ILRs in simulated burned samples.

## 2. Experimental Section

### 2.1. Samples

Burned samples were prepared in the laboratory. For this purpose, six substrate samples (pine wood, cork, paper, newspaper, cardboard, and cotton sheet) were burned without any accelerant and then with each of six ignitable liquids (gasoline, diesel, kerosene, citronella, paraffin, and ethanol). Both the substrates and ignitable liquids were purchased in local Spanish stores.

Burned samples were denoted by the a liquid code followed by the substrate code, for instance, *Nco* for cork burned alone, *Gpw* for pine wood burned with gasoline and so on (Table 1).

To simulate post burn samples, the method by the U.S. National Center for Forensic Science was used with some minor changes [4]. Laboratory fire debris samples were generated as follows: using the solid materials listed in Table 1, 500 µL of different ignitable liquids were added. The samples were placed upside down in an unlined one-quart paint can and covered with a vented lid with nine 1 mm diameter holes. Heat was applied to the bottom of the can by a flame from a propane torch held at a distance 4 cm from the bottom. The contents of the can was allowed to burn for two additional minutes after smoke appeared. Then, the can was allowed to cool down to room temperature. To avoid losing the volatile compounds from the headspace, the perforated lid was replaced with an intact lid. Once the can was cool, the burned samples were ready for analysis. After burning samples were transferred from the paint cans to 10 mL vials to obtain the MS spectra.

### 2.2. Acquisition of E-Nose Spectra

Analysis of the samples was performed on an Alpha Moss (Toulouse, France) E-Nose system composed of an HS 100 static headspace auto sampler and a Kronos quadrupole mass spectrometer (MS). The samples were contained in 10 mL sealed vials (Agilent Crosslab, Santa Clara, CA USA), which were placed in the autosampler oven to be heated and agitated in order to generate the headspace. Headspace was taken from the vial using a gas syringe and injected into the mass spectrometer. Between each sample injection, the gas syringe was flushed with carrier gas (nitrogen) to avoid cross-contamination.

The optimized experimental conditions for the headspace sampler were as follows: incubation temperature 115 °C, incubation time 10 min, agitation speed 500 rpm, syringe type 5 mL, syringe temperature 150 °C, flushing time 120 s, fill speed 100 µL/s, injection volume 4.5 mL, and injection speed 75 µL/s. The carrier gas was nitrogen. The total time per sample was approximately 12 min.

The components in the headspace of the vials were passed directly to the mass detector without any chromatographic separation or sample pre-treatment. In this way, for any given measurement, the resulting mass spectrum gives a fingerprint of the sample. Electron ionization spectra were recorded in the range *m*/*z* 45–200. Instrument control was achieved using the Residual Gas Analysis (RGA) software package and the Alpha Soft 7.01 software.

### 2.3. Data Analysis and Software

All of the MS data were normalized to the base peak at 100%. Multivariate analysis of the data, which included hierarchical cluster analysis (HCA) and linear discriminant analysis (LDA), was performed using the statistical computer package SPSS 17.0 (SPSS Inc., Chicago, IL, USA).

## 3. Results and Discussion

### 3.1. Fire Debris Samples

A set of similar burned samples was created for the optimization process. Only one type of substrate was used for the optimization process, namely pine wood sticks, which were separately burned using gasoline (*Gpw*), diesel (*Dpw*), and citronella (*Cpw*) as accelerants. The three different ignitable liquids were chosen for the optimization process in order to ensure that the resulting optimized method is applicable to different types of ignitable liquids. Gasoline and diesel are chemically different and they are the ignitable liquids that are most commonly used by arsonists. On the other hand, citronella was chosen to ensure that the method is also able to discriminate *vs*. other types of accelerants.

With the aim of ensuring that all of the burned samples contained the same ILRs, different sets of burning experiments with each liquid were performed and the burned pine wood sticks from the different sets (but same ignitable liquid) were mixed, thus decreasing heterogeneity.

### 3.2. Headspace Generation. Optimization of the Working Conditions

The experimental conditions that affect the headspace generation process were studied and those that can be controlled for fire debris samples are incubation temperature and incubation time. The sample volume may also affect the headspace, but in real fire debris samples the quantity of ignitable liquid residue that remains in the substrate cannot be known. The volume was therefore not optimized. A total of four wood sticks were burned in each can. Later they were randomly used for the analysis. The samples were directly analyzed by E-Nose without an isolation step for the ILRs. In this case the pre-concentration of the ILRs takes place during the headspace generation itself.

**Figure 1:**
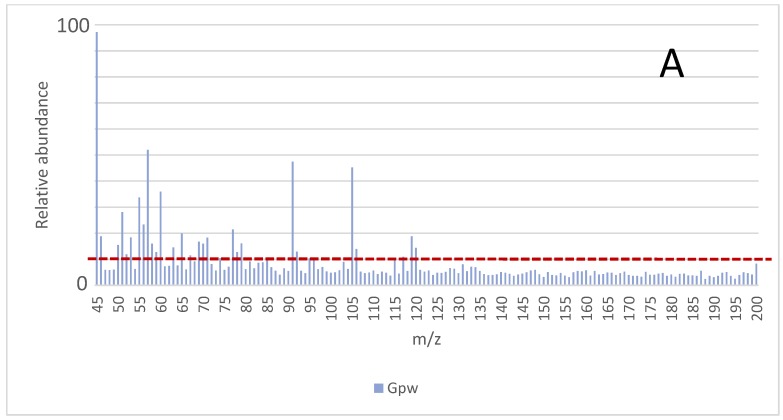

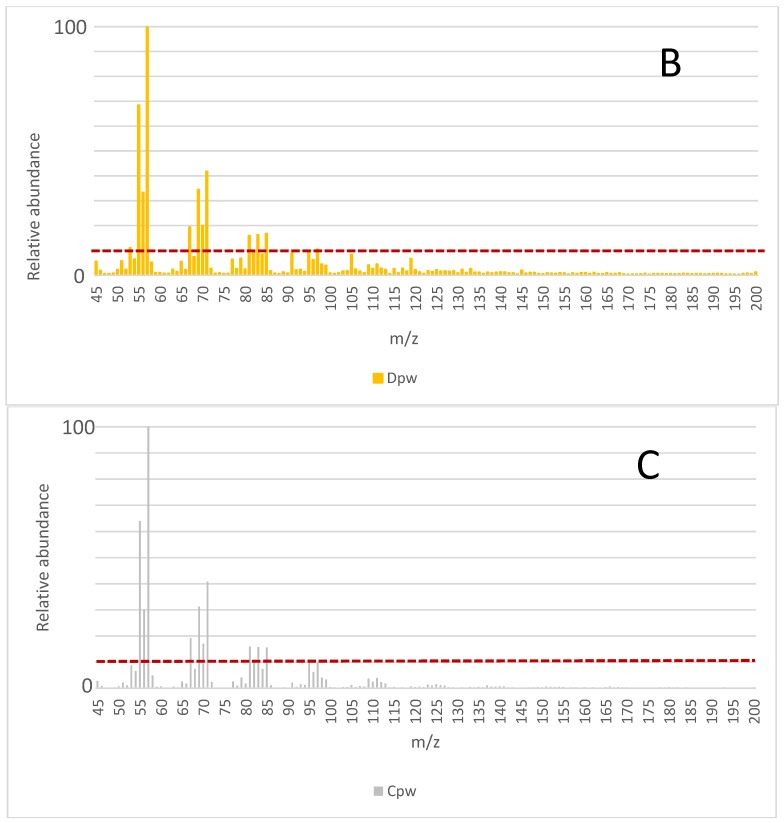
Spectra for the burned samples: *Gpw* (**A**); *Dpw* (**B**); and *Cpw* (**C**). Average values for all assayed incubation temperatures and incubation times. The *m*/*z* values with an intenisty above the red line were considered for the optimization.

The results obtained under the different experimental conditions, *i.e.*, incubation temperature and incubation time, were compared by considering the signals (*m*/*z*) from the MS that had at least 10% of the intensity of the maximum abundance from the mass spectrum. That value (10%) was indicated with the red dotted line in Figure 1. All analyses were carried out in duplicate and the average mass values are presented. The average spectra obtained for the samples *Gpw*, *Dpw*, and ***Cpw*** are shown in Figure 1 and the *m*/*z* values that comply with this requirement under the different conditions are indicated. 

**Figure 2:**
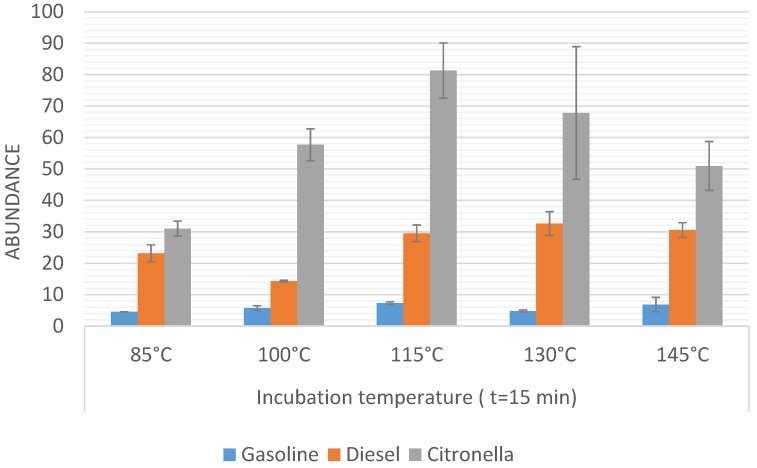
Sum abundance values obtained for the burned samples *Gpw*, *Dpw*, and *Cpw*. Heating time 15 min at different incubation temperatures. Bars with the same letter for the same ILR indicate a non-significant difference (*p* < 0.05) according to ANOVA analysis.

Firstly, the incubation temperature was optimized by fixing the incubation time at 15 min and comparing the results obtained on applying different incubation temperatures (range from 85 °C to 145 °C). The time of 15 min was selected on the basis of results obtained in previous experiments on the same system. The results obtained in the temperature optimization for the three sample sets are represented in Figure 2. Samples were run in triplicate, then the average values and the resulting standard deviation for the total sum of abundances were used to be compared. The bars in Figure 2 represent the sum of the abundance for those *m*/*z* signals that are higher than 10% of the maximum signal for each sample. An ANOVA study was carried out in order to check if the differences between the different methods were statistically significant.

It can be observed that the maximum signal for the gasoline ILR (*Gpw* samples) was obtained when the samples were heated for 15 min at 115 °C. Significant differences (F < Fcrit) were not observed when the temperature was increased above 115 °C, although there was a significant difference at lower temperature. For an incubation temperature of 85 °C, the signal decreased by 40%.

In the case of *Dpw* samples, the maximum signal was obtained at 130 °C but this did not show significant differences from the results obtained at 115 °C or 145 °C. In contrast, incubation temperatures of less than 100 °C did decrease the signal significantly. It can be seen that a decrease in the temperature from 115 °C to 85 °C led to a decrease in the sum abundance by 50%.

Samples burned with citronella (*Cpw* samples) showed a maximum signal at 115 °C. However, in this case a significant decrease in the signal was not observed at a temperature of 100 °C, but there were clear differences for temperatures below 100 °C. An incubation temperature of 80 °C led to a decrease in the signal by 62%. 

Based on these results, 115 °C was chosen as the optimal temperature since significant differences were not observed for *Dpw vs.* higher temperatures, whilst it was the highest signal for *Cpw* samples. However, in the case of *Gpw* a significant difference was found between 115 °C and 100 °C.

**Figure 3:**
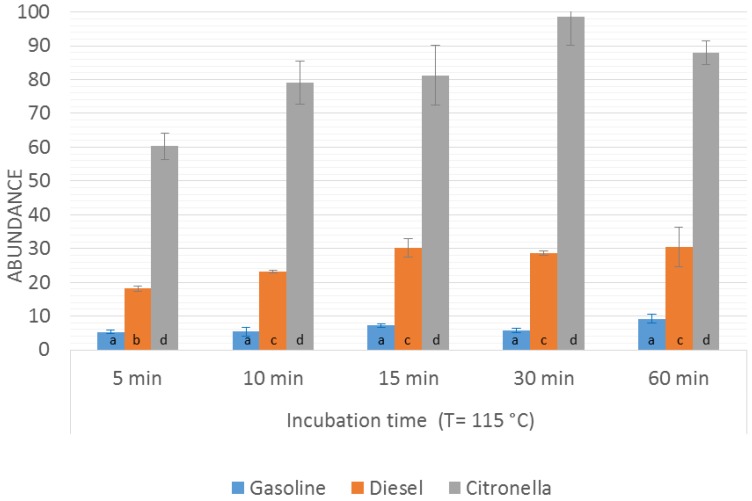
Sum abundance values obtained for the burned samples *Gpw*, *Dpw*, and *Cpw*. Incubation temperature 115 °C and different incubation times. Bars with the same letter for the same ILR indicate a non-significant difference (*p* value < 0.05) according to ANOVA analysis.

Once the optimal incubation temperature had been selected, the optimization of the incubation time was addressed by fixing the incubation temperature at 115 °C and comparing the results obtained on applying different incubation times (range from 5 min to 60 min). The *m*/*z* values selected for the comparison were again the sum of abundances for *m*/*z* signal that were at least 10% of the maximum one (Figure 1). The results obtained in the time optimization for the burned samples *Gpw*, *Dpw*, and *Cpw* are presented in Figure 3.

In the case of *Gpw* and *Cpw* samples, similar results were obtained regardless of the incubation time, meaning that the equilibrium in the headspace is reached after a few minutes and that an increase in the incubation times does not enhance the signal in a significant way.

As far as *Dpw* samples are concerned, it was observed that an increase in the incubation time led to an increase in the signal up to 15 min, but after this time the intensity of the signal remained almost constant. No significant difference was seen between the results obtained at 10 min and 15 min, although significant differences were apparent for an incubation time of 5 min, with a decrease of almost 40% in the signal. This result could be related to the fact that diesel contains more heavy compounds and it requires longer times at the given temperature to reach equilibrium in the headspace. Based on these results, 10 min was selected as the optimal incubation time.

### 3.3. Applicability in the Discrimination of Different Types of Fire Debris

A set of different burned samples was created in order to assess the applicability of the optimized method in the discrimination of different ILRs from fire debris according to the ignitable liquids used as accelerants. Different substrates (pine wood, cork, paper, newspaper, paperboard, and cotton sheet) and ignitable liquids (gasoline, diesel, kerosene, citronella, paraffin, and ethanol) were used. The samples were denoted as shown in Table 1. All samples were analyzed in duplicate.

In order to assess the tendency of the different burned samples to cluster, an exploratory chemometric technique was initially applied. HCA was applied by using all of the *m*/*z* (45−200 *m*/*z*) values obtained from the E-Nose as a variable to form groups. All spectra were normalized to the base peak at 100%.

**Figure 4:**
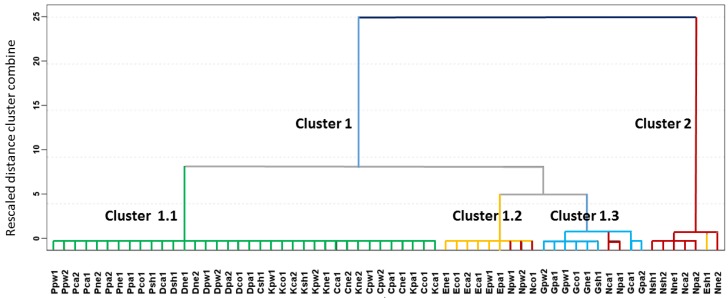
Dendrogram obtained from HCA for fire debris samples (*n* = 63) using the MS data from E-Nose (45–200 *m*/*z*).

The results of the HCA are represented in the dendrogram in Figure 4, in which all of the samples are listed and the level of similarity (dissimilarity) at which any of the two clusters were joined is indicated. The Ward method was used for cluster preparation and square Euclidean distance was used to measure distances between clusters.

A tendency to cluster the samples according to the ignitable liquid used as an accelerant was found on applying HCA. It can be observed that there are two large clusters, namely Clusters 1 and 2. Cluster 1 contains all of the substrates burned with accelerant except for one sample burned with ethanol (*Esh1*), which was classified in Cluster 2. The second cluster contains the substrates burned alone. Furthermore, Cluster 1 is divided into three sub-groups. Cluster 1.1 includes all the substrates burned with heavy ignitable liquids such as paraffin, diesel, kerosene, and citronella. Cluster 1.2 includes six of the seven samples burned with ethanol and three burned without an IL. Cluster 1.3 contains all of the samples burned with gasoline and two burned without IL.

The results discussed above indicate that the data from the E-Nose analyses used to perform the HCA are related to the compounds that are responsible for the discrimination of the fire debris according to the ignitable liquids used to burn them, even though the 155 signals obtained from the E-Nose were not sufficient to obtain a perfect separation of all analyzed samples. No further improvements in the HCA results were studied because of the main interest was related to a mathematical function allowing for an automatic classification of the samples.

Given that the non-supervised technique suggested some kind of classification, a supervised technique, namely Linear Discriminant Analysis (LDA), was applied to the whole body of mass spectra. In order to obtain a more robust discrimination prior to running the LDA, 75% (*n* = 47) of the samples were randomly selected as a training set in order to obtain discriminant functions and the remaining 25% (*n* = 15) of the samples were then used as a validation set. A stepwise discriminant analysis was applied in order to identify whether there are specific *m*/*z* values in the mass spectra that are more significant than the others when classifying the ILRs.

The resulting discriminant function allowed a full discrimination between the seven groups of samples. The *m*/*z* values selected to develop the discrimination function were as follows: *m*/*z* 49, *m*/*z* 57, *m*/*z* 60, *m*/*z* 67, *m*/*z* 69, *m*/*z* *m*/*z* 81, *m*/*z* 84, *m*/*z* 85, *m*/*z* 90, *m*/*z* 91, *m*/*z* 96, *m*/*z* 121, *m*/*z* 142, *m*/*z* 154, *m*/*z* 176 and *m*/*z* 196, meaning that these *m*/*z* values are related to the ILR in fire debris that produces the final mass spectra. The samples in both the calibration and validation sets were clearly assigned to their corresponding group without exception, *i.e.*, 100% correct classification was obtained for all samples.

**Figure 5:**
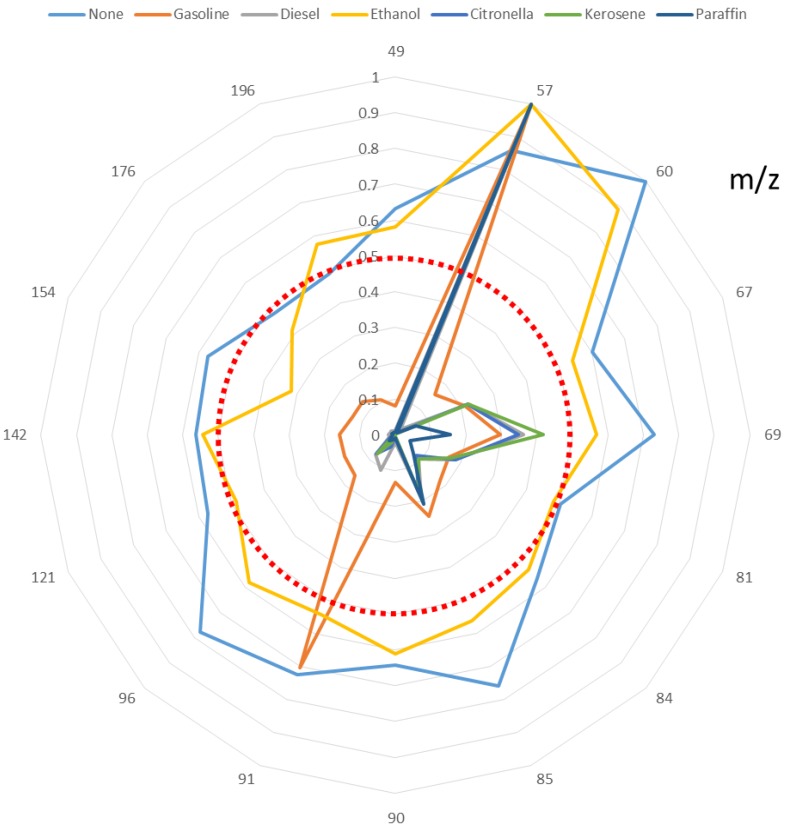
ILR fingerprints obtained by displaying the *m*/*z* values selected in the LDA. The red line represents 50% of the highest value.

The *m*/*z* values selected to develop the discriminant function for each ignitable liquid residue are shown in Figure 5. All of the selected *m*/*z* values have been normalized to the base peak at 100%. As can be seen, the fingerprint created by each ILR is different. Only samples burned without IL or with ethanol give rise to *m*/*z* values above 50% of the maximum intensity. Samples burned with gasoline presented two *m*/*z* (*m*/*z* 57 and *m*/*z* 91) with values above 50% of the highest value. Samples burned with heavy ignitable liquids present only one *m*/*z* above 50% of the highest value, and this is the same for all such samples (*m*/*z* 57). However, the intensities and the ratios of the rest of the signals are different for each ILR, thus giving different fingerprints that can be used for the discrimination of the different ILRs.

## 4. Conclusions

The E-Nose has proven to be a promising tool and it can be considered a promising alternative for further exploration for the analysis of accelerants in arson investigations. This technique has several advantages over the reference method. First, the E-Nose is much faster than the ASTM E1412 reference method. Second, E-Nose does not require any solvent, so it is safer for the user and is ecofriendly. Third, the E-Nose is cheaper since it does not require any absorbent or solvent. Finally, the E-Nose is easy to use in routine analysis and it does not require a specially trained analyst. In order to make a more robust method, some additional samples should be evaluated, including several gasoline and diesel samples from other regions and countries, to include differences due to different brands and processing conditions.

## Figures and Tables

**Table 1:** Ignitable liquids and substrates used for the preparation of burned samples.

Ignitable Liquid	Code IL	Substrate	Code Substrate
None	N	pine wood	pw
Gasoline	G	cork	co
Diesel	D	paper	pa
Ethanol	E	newspaper	ne
Citronella	C	cardboard	ca
Kerosene	K	sheet	sh
Paraffin	P		
